# Supplementary material for: Causal Relationship between Aging and Anorexia Nervosa: A White-Matter-Microstructure-Mediated Mendelian Randomization Analysis
Source: Biomedicines. 2024 Aug 16;12(8):1874. doi: 10.3390/biomedicines12081874 (PMC11351342; doi:10.3390/biomedicines12081874)
Supplement: Supplementary file 1 [file biomedicines-12-01874-s001.zip › Supplementary Figure/Figure S4/Singe SNP plot of MR analysis for TL to ubm-b-1837.pdf]

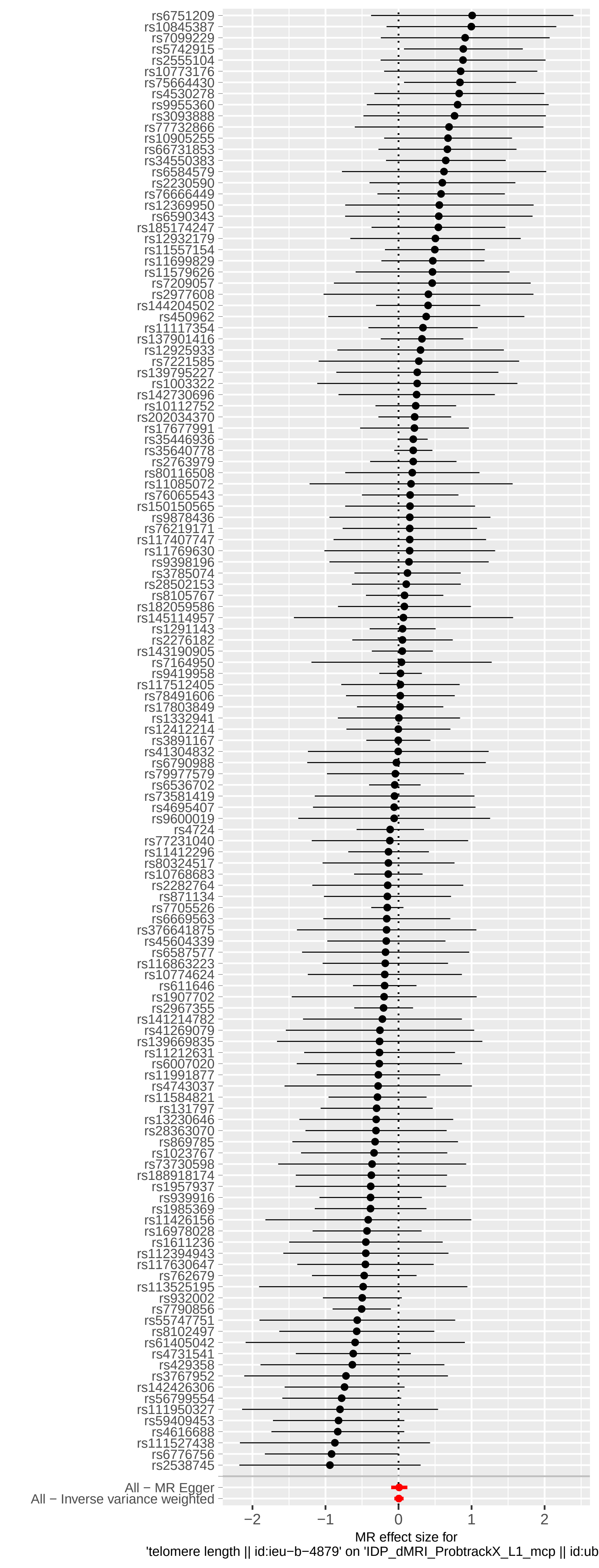

rs6751209
rs10845387
rs7099229
rs5742915
rs2555104
rs10773176
rs75664430
rs4530278
rs9955360
rs3093888
rs77732866
rs10905255
rs66731853
rs34550383
rs6584579
rs2230590
rs76666449
rs12369950
rs6590343
rs185174247
rs12932179
rs11557154
rs11699829
rs11579626
rs7209057
rs2977608
rs144204502
rs450962
rs11117354
rs137901416
rs12925933
rs7221585
rs139795227
rs1003322
rs142730696
rs10112752
rs202034370
rs17677991
rs35446936
rs35640778
rs2763979
rs80116508
rs11085072
rs76065543
rs150150565
rs9878436
rs76219171
rs117407747
rs11769630
rs9398196
rs3785074
rs28502153
rs8105767
rs182059586
rs145114957
rs1291143
rs2276182
rs143190905
rs7164950
rs9419958
rs117512405
rs78491606
rs17803849
rs1332941
rs12412214
rs3891167
rs41304832
rs6790988
rs79977579
rs6536702
rs73581419
rs4695407
rs9600019
rs4724
rs77231040
rs11412296
rs80324517
rs10768683
rs2282764
rs871134
rs7705526
rs6669563
rs376641875
rs45604339
rs6587577
rs116863223
rs10774624
rs611646
rs1907702
rs2967355
rs141214782
rs41269079
rs139669835
rs11212631
rs6007020
rs11991877
rs4743037
rs11584821
rs131797
rs13230646
rs28363070
rs869785
rs1023767
rs73730598
rs188918174
rs1957937
rs939916
rs1985369
rs11426156
rs16978028
rs1611236
rs112394943
rs117630647
rs762679
rs113525195
rs932002
rs7790856
rs55747751
rs8102497
rs61405042
rs4731541
rs429358
rs3767952
rs142426306
rs56799554
rs111950327
rs59409453
rs4616688
rs111527438
rs6776756
rs2538745
All − MR Egger
All − Inverse variance weighted
−2
−1
0
1
2
MR effect size for
'telomere length || id:ieu−b−4879' on 'IDP_dMRI_ProbtrackX_L1_mcp || id:ub
